# Baltikinin: A New Myotropic Tryptophyllin-3 Peptide Isolated from the Skin Secretion of the Purple-Sided Leaf Frog, *Phyllomedusa baltea*

**DOI:** 10.3390/toxins8070213

**Published:** 2016-07-08

**Authors:** Daning Shi, Xinping Xi, Lei Wang, Yitian Gao, Chengbang Ma, Hang Chen, Mei Zhou, Tianbao Chen, Chris Shaw

**Affiliations:** 1 Natural Drug Discovery Group, School of Pharmacy, Queen’s University, Belfast BT9 7BL, Northern Ireland, UK; dshi01@qub.ac.uk (D.S.); x.xi@qub.ac.uk (X.X.); l.wang@qub.ac.uk (L.W.); ygao07@qub.ac.uk (Y.G.); c.ma@qub.ac.uk (C.M.); t.chen@qub.ac.uk (T.C.); chris.shaw@qub.ac.uk (C.S.); 2 Fujian Provincial Institute for Food and Drug Quality Control, Fuzhou, Fujian 350000, China

**Keywords:** amphibian, peptide, smooth muscle, molecular cloning

## Abstract

Here we report the identification of a novel tryptophyllin-3 peptide with arterial smooth muscle relaxation activity from the skin secretion of the purple-sided leaf frog, *Phyllomedusa baltea*. This new peptide was named baltikinin and had the following primary structure, pGluDKPFGPPPIYPV, as determined by tandem mass spectrometry (MS/MS) fragmentation sequencing and from cloned skin precursor-encoding cDNA. A synthetic replicate of baltikinin was found to have a similar potency to bradykinin in relaxing arterial smooth muscle (half maximal effective concentration (EC_50_) is 7.2 nM). These data illustrate how amphibian skin secretions can continue to provide novel potent peptides that act through functional targets in mammalian tissues.

## 1. Introduction

Amphibian skin secretions have provided a wide range of bioactive peptides for international biochemical and pharmacology research communities [1,2,3,4]. Improvements in techniques of identification and classification have led to the discovery of increasing numbers of such biologically active peptides. The structures and functional activities of hundreds of biogenic amines and active peptides from amphibian skin were originally presented by Vittorio Erspamer, a leading pioneer in the field for more than half a century [4,5,6]. Erspamer predicted that many of the peptides found in amphibian skin secretions would have endogenous regulatory counterparts in mammalian tissues—a prediction that has proven to be true [4,5,6]. Following these early studies, research has been performed on over 200 American amphibian species as well as hundreds of species from other continents. Initial studies were based on identification of novel structures by their physiological/pharmacological actions and, as a consequence, many were overlooked [4,5,6,7,8,9]. Since then, it has been established that these skin compounds secreted by amphibians may have regulatory functions in their physiological systems and certainly act in defense against predators or invading microorganisms [4,5,6,7]. Systematic structural analysis of these skin-derived bioactive peptides has enabled researchers to assign individual peptides to previously identified families on the basis of their amino acid sequence similarities [4,8,9]. Functional genomics has been used recently as an approach to establish their genetic coding and precursor structures, primary structures and pharmacological activities as previously described. This approach has been employed to assign biological roles and activities to a wide range of peptides from various species. In the meantime, the combination of functional genomics and structural data, which are produced from biosynthetic precursor cDNA cloning and functional target studies, can provide a holistic approach to the investigation of natural bioactive peptides [10,11,12,13,14,15,16,17].

The neotropical frogs, of the subfamily Phyllomedusinae, have the highest peptide molecular complexity in their skin secretions among the amphibians [6,7]. The subfamily Phyllomedusinae is further sub-divided into three major genera, namely *Phyllomedusa,* *Agalychnis*, and *Pachymedusa*. However, this has been revised in recent years to seven genera, namely *Agalychnis*, *Cruziohyla*, *Hylomantis*, *Pachymedusa*, *Phasmahyla*, *Phrynomedusa*, and *Phyllomedusa* [18]. The *Phyllomedusa* genus is a diverse taxon of hylid frogs with a wide range of skin peptides, many of which have been grouped into structurally-related families [5,6,7]. The presence of highly-conserved signal peptides in their biosynthetic precursor proteins is suggestive of a similar phylogenetic ancestry [19,20,21]. Peptides that have been isolated from these frogs include tachykinins, bradykinins, caeruleins, bombesins, sauvagine, opioid peptides, antimicrobial peptides and tryptophyllins, amongst others. Previous studies on peptides from skin secretions within this subfamily mainly focused on those isolated from the genus *Phyllomedusa* such as the species *P. sauvagei*, *P. burmeisteri*, *P. rohdei*, and *P. bicolor*. This is unsurprising when it is considered that this genus is the largest within this subfamily [18].

Here, we describe the structural and functional characterization of a novel myotropic tryptophyllin-3 peptide, named baltikinin, from the skin secretion of the purple-sided leaf frog, *Phyllomedusa baltea*. The baltikinin precursor, deduced from cloned skin cDNA, consisted of 63 amino acid residues with a single copy of baltikinin located near the *C*-terminus. Baltikinin is an *N*-terminally pyroglutamylated peptide with the following mature peptide primary structure: pGluDKPFGPPPIYPV. It was found to relax the smooth muscle of rat tail artery with an EC_50_ of 7.2 nM.

## 2. Results

### 2.1. Molecular Cloning of Baltikinin Precursor-Encoding cDNA from a Skin Secretion-Derived cDNA Library of Phyllomedusa Baltea

**Figure 1:**
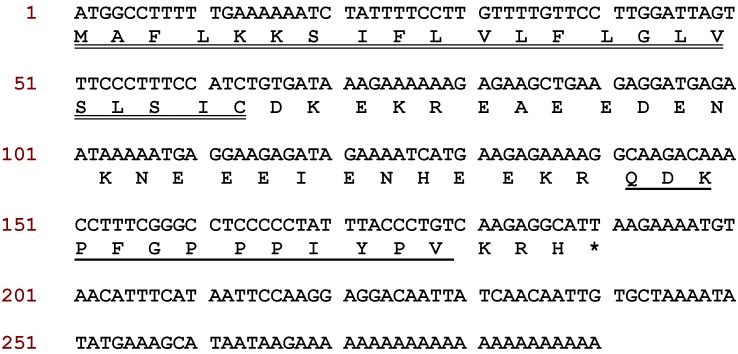
Nucleotide and translated open reading frame amino acid sequence of baltikinin precursor encoding cDNA from the skin secretion of the purple-sided leaf frog, *Phyllomedusa baltea*. The putative signal peptide sequence is double-underlined, the mature peptide sequence is single-underlined and the stop codon is indicated by an asterisk.

**Figure 2:**
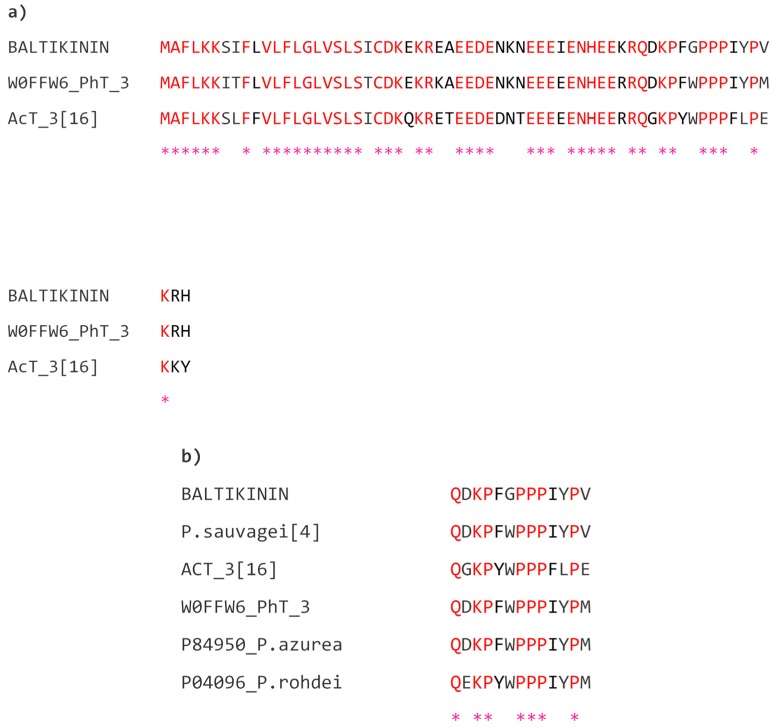
Alignments of significant related amino acid sequences found in the NCBI database: (**a**) the alignment of related peptide precursors from three members of the Phyllomedusinae subfamily; and (**b**) the alignment of mature related peptides from six members of the Phyllomedusinae subfamily. Sites of identical sequence are indicated by asterisks.

One cDNA was consistently cloned and sequenced from the skin secretion cDNA library of *Phyllomedusa baltea* using the “shotgun” cloning strategy described and this encoded the open reading frame of a peptide precursor containing a novel bioactive peptide which was subsequently named baltikinin (Figure 1). The open-reading frame consisted of a putative 22-m *N*-terminal signal peptide (a short peptide chain that directs the post-translational transport of a protein) followed by an acidic “spacer” peptide which terminated in a typical -Lys-Arg-(-KR-) propeptide convertase processing site. This site is responsible for the enzymatic cleavage/release of the *N*-terminus of the mature peptide. At the *C*-terminus, the mature baltikinin peptide was generated by removal of three terminal amino acid residues. Bioinformatic investigations were performed using the BLAST search of structural similarity through the National Centre for Biotechnological Information (NCBI) web portal. Figure 2A shows that this precursor exhibited high amino acid sequence similarity to tryptophyllin type-3 (T3) peptides from other *Phyllomedusa* frogs [16,17]. The NCBI-BLAST search also showed that the mature peptide exhibited a high degree of structural identity with homologous peptides from other species of Phyllomedusinae (Figure 2B) [4].

### 2.2. Identification and Structural Analysis of Baltikinin

**Figure 3:**
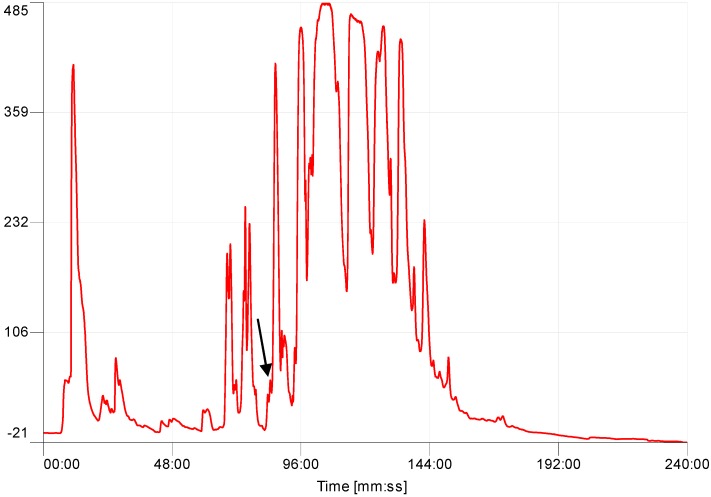
Reversed-phase HPLC chromatogram of skin secretion from the purple-sided leaf frog, *Phyllomedusa baltea,* with an arrow indicating the elution position/retention time of baltikinin.

**Figure 4:**
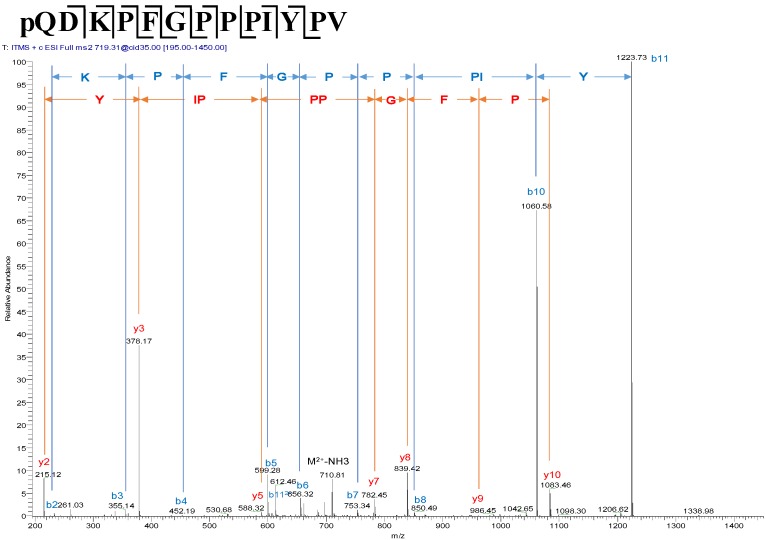
Tandem mass spectrometry (MS/MS) spectrum of the predicted peptide, baltikinin, in the HPLC fraction. Series of *b* (blue) and *y* (red) ions of the candidate peptide were observed. The amino acid sequence is shown at the top of the spectrum.

Many components of the skin secretion were resolved following reverse-phase HPLC fractionation. The retention time/elution position of the putative mature peptide, baltikinin, was identified by use of its computed molecular mass from cloned precursor-encoding cDNA in the interrogated data sets generated by Matrix-assisted laser desorption/ionisation, time-of-flight Mass spectrometry (MALDI-TOF MS) analyses. The mature peptide of coincident molecular mass was observed in the peak eluted at 85 min (Figure 3). The confirmation that this peptide was the predicted peptide, baltikinin, was obtained through primary structural assignation using MS/MS fragmentation sequencing (Figure 4).

### 2.3. Peptide Synthesis

The solid phase peptide synthesis of baltikinin was successfully accomplished using the PS3 automated synthesizer. The crude product was subjected to reversed-phase HPLC and this indicated that the synthetic peptide was of insufficient purity for subsequent functional studies (data not shown). However, it was further purified to >95% purity using HPLC. This was confirmed by MALDI-TOF analysis (data not shown).

### 2.4. Arterial Smooth Muscle Pharmacology

**Figure 5:**
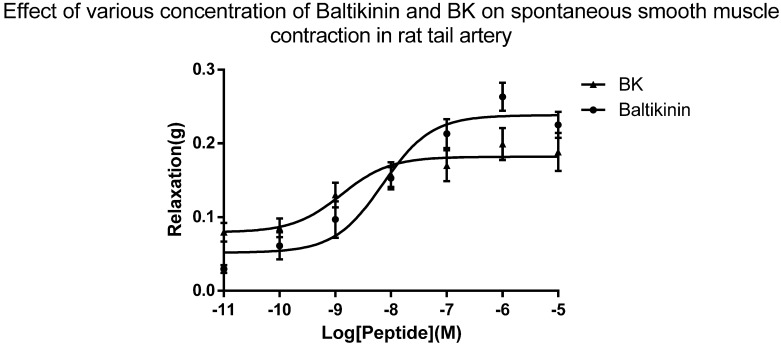
Dose-response curve of baltikinin on rat tail arterial smooth muscle preparations after phenylephrine (1 mM) pre-contraction. Each point was plotted as negative tension changes and represents the mean ± SEM (*n* = 7). The EC_50_ values of baltikinin and BK were 7.16 nM and 1.29 nM, respectively.

Dose–response curves were constructed for the peptide and bradykinin (BK), as a standard, on individual arterial smooth muscle preparations (*n* = 7). BK was no exception in processing relaxation on the precontracted rat tail arterial. Moreover, baltikinin exhibited BK-like activity on the same preparations. The calculated EC_50_ of baltikinin and BK were 7.16 nM and 1.29 nM, respectively (Figure 5).

## 3. Discussion

Many novel peptides from amphibian skin secretions have been characterized based on chemical attributes, structure, activity in bioassays and functionality [1,2,3,4]. Pharmacological studies allow peptides to be classified structurally and functionally and offer a more holistic approach in determining the biological role/activity of novel peptides often overlooked by previously published work. More specifically, molecular cloning and peptide sequencing techniques employed in parallel for identification of bioactive peptides have been developed for the confirmation of peptide structures [8,9,10,11,12,13,14,15,16,17].

It is well acknowledged throughout the published literature, that many of the peptides from amphibian skin secretions have profound myotropic, antimicrobial and hemolytic activities [4]. Through a much needed deeper understanding of pharmacological actions, phylogenetic relationships, taxonomy and structural similarities, identification of biological roles for these peptides will allow progression in the search for therapeutically useful applications [22].

Here, we identified a novel myotropic bioactive peptide and its biosynthetic precursor from skin secretion of the purple-sided leaf frog, *Phyllomedusa baltea*. This was the first time such a peptide had been cloned and characterized from this species. The mature peptide sequence was used to perform a database search using NCBI-BLAST and the result showed that this shared a highly-conserved sequence in an internal -PPPIYP- motif of T3-tryptophyllin tridecapeptides (Figure 2B). It was speculated that T3-tryptophyllins could have the biological activity of relaxation of blood vessels, as they share the same motifs, -PP-, -PPP- and KP-, with hypotensins (TsHpt) reported from the venom of the Brazilian scorpion, *Tityus serrulatus* [23]. However, previously, known T3-tryptophyllins were investigated by Erspamer’s group for such an effect but were found to be inactive in smooth muscle assays using various tissue preparations [4].

The novel peptide here described, demonstrated a highly-conserved sequence with other T3-tryptophyllin peptides, including a pyroglutamic acid at the *N*-terminus and an identical internal sequence motif, but possessed a glycyl residue at position 6. As has been documented before, the T3-tryptophyllins identified to date, display a double aromatic amino acid motif, -PF/YW/YPPP-, which could be significant for their biological activity. Therefore, we proposed that the substitution of the second aromatic residue in this motif with glycine, might affect biological function and performed a pharmacological assay utilizing a rat tail artery preparation. In this, the novel peptide displayed a potent bradykinin-like activity in relaxing the smooth muscle with an EC_50_ of 7.16 nM, which is similar to bradykinin (EC_50_: 1.29 nM). In contract to the T3-tryptophyllin peptide studied by Erspamer [4], two other highly-conserved T3-tryptophyllin peptides, -PhT-3 and AcT-3, both of which contain a double aromatic amino acid motif, demonstrated non-competitive inhibition against bradykinin-induced relaxation of rat arterial smooth muscle [16,17]. These data collectively suggested that the lack of the double aromatic amino acid motif is likely to be responsible for these contrary activities, owning to the replacement of Trp by Gly at position 6 of baltikinin. Due to its novel primary structure and potent novel bioactivity, we considered that this Gly^6^-T3-tryptophyllin should be given a unique name—baltikinin, in which the “balti-” was derived from the frog specific name *baltea* and (“-kinin”) to denote its similar activity to bradykinin.

Although, baltikinin induced relaxation of phenylephrine pre-contracted rat artery smooth muscle, the mechanism of action was not examined in detail. Theoretically, the aromatic residue at position 6 may significantly contribute to this biological activity [24]. This assumption is based on structure/activity studies of bradykinin, which indicated a key role of Phe in its sequence. It was reported that bradykinin was converted to a bradykinin receptor antagonist by changing Phe, an aromatic amino acid residue, to Leu, a non-aromatic amino acid residue, at position 8 [24]. Thus, in this case, another aromatic amino acid residue, Trp, was replaced by Gly, which may change the ligand-receptor interaction and alter the related signaling pathway, thus altering biological activity.

It is the first report of a potent myoactive T3-tryptophyllin, named baltikinin, from skin secretion of a phyllomedusine tree frog. Further investigation of the molecular target of this peptide, may pave the way for development of novel therapies relating to hypertension.

## 4. Materials and Methods

### 4.1. Skin Secretions

Specimens of *Phyllomedusa baltea* were collected in Peru by PeruBiotech E.I.R.L. The skin secretion (40 mg lyophilized dry weight) of captured adults was subsequently harvested using mild electrical stimulation of the dorsal skin surface. Briefly, the moistened skin was stimulated by platinum electrodes (6 V DC; 4 ms pulse-width; 50 Hz) for two periods of 20 s duration. After this, stimulated secretion was collected by washing from the frog skin using distilled, deionized water. These skin secretions were snap-frozen in liquid nitrogen, lyophilized and stored at 20 °C prior to analysis. Sampling of skin secretion was performed by Mei Zhou under UK Animal (Scientific Procedures) Act 1986, project license PPL 2694, issued by the Department of Health, Social Services and Public Safety, Northern Ireland. Procedures had been vetted by the IACUC of Queen's University Belfast, and approved on 1 March 2011.

### 4.2. “Shotgun” Cloning of Preprobaltikinin Encoding cDNA

Polyadenylated mRNA was isolated from the stabilization buffer using magnetic oligo-dT beads as described by the manufacturer (Life technologies, Oslo, Norway) and reverse-transcribed. The cDNA was subjected to 3′-RACE procedures to obtain full-length prepropeptide nucleic acid sequence data using a SMART-RACE kit (Clontech, Palo Alto, CA, USA) essentially as described by the manufacturer. Briefly, the 3′-RACE reactions employed a NUP primer (supplied with the kit) and a degenerate sense primer (S1; 5′-ACTTTCYGAWTTRYAAGMSCARABATG-3′) that was designed to a highly conserved domain of the 5′-untranslated region of previously characterized antimicrobial peptide cDNAs from members of the *Phyllomedusa* genus [10,11,12,13,14,15,16,17,21]. The PCR cycling procedure was as follows. Initial denaturation step: 60 s at 94 °C; 35 cycles: denaturation 30 s at 94 °C, primer annealing for 30 s at 58 °C; extension for 180 s at 72 °C. PCR products were gel-purified, cloned using a pGEM-T vector system (Promega Corporation, Southampton, UK), at 72 °C. PCR products were gel-purified and cloned using a pGEM-T vector system and sequenced using an ABI 3100 automated sequencer (Applied Biosystems, Foster City, CA, USA).

### 4.3. Identification and Structural Characterisation of Baltikinin from the Skin Secretion

Lyophilized skin secretion (5 mg) was dissolved in 1 mL of TFA/water (0.05/99.95, *v*/*v*). The samples were centrifuged in an Eppendorf Centrifuge 5418 (Eppendorf, Hamburg, Germany) at 2500 g for 10 min to remove particulate matter. The supernatant was fractionated by injecting directly onto a reverse phase HPLC column (Jupiter C-5, 250 mm × 10 mm, Phenomenex, Macclesfield, Cheshire, UK) and peptides were eluted using a gradient formed from 0.05/99.5 (*v*/*v*) TFA/water to 0.05/19.95/80.0 (*v*/*v*/*v*) TFA/water/acetonitrile in 240min at a flow rate of 1 mL/min. A Cecil CE4200 Adept (Cecil Instruments Limited, Cambridge, Cambridgeshire, UK) gradient reverse phase HPLC system was employed and fractions were collected automatically at 1 min intervals. 1 µL of each chromatographic fraction was prepared for mass analysis using MALDI-TOF mass spectrometry on a linear time-of-flight Voyager DE mass spectrometer (Voyager DE, Perseptive Biosystems, Foster City, CA, USA) in positive detection mode using alpha-cyano-4-hydroxycinnamic acid as the matrix. Internal mass calibration of the instrument was achieved using standard peptides of established molecular mass providing a determined accuracy of ±0.1%. Peptides in the molecular mass range of baltikinin (around 1.44 kDa) were each subjected to primary structural analysis by MS/MS fragmentation sequencing using an LCQ-Fleet electrospray ion-trap mass spectrometer (Thermo Fisher Scientific, San Francisco, CA, USA).

### 4.4. Peptide Synthesis

Baltikinin was synthesized automatically using the solid-phase method (Wang-resin, 0.6 mmol/g, Calbiochem-Novabiochem AG, Läufelfingen) and 9 standard fluorenylmethoxycarbonyl (Fmoc) chemistry (double couplings with 8 equivalent of Fmoc-amino acid derivatives) using a PS3 automated solid phase peptide synthesizer (Protein Technologies, Tucson, AZ, USA). Couplings were performed using 2-(1H-benzotriazol-1-yl)-1,1,3,3-tetramethyluronium tetrafluoroborate (Calbiochem-Novabiochem AG, Läufelfingen).

Solid phase peptide synthesis begins with the covalent attachment (via its carboxyl group) of the *C*-terminal amino acid to an insoluble particle such as a polystyrene resin. The *N*-terminal end of the bound peptide undergoes coupling with carboxyl-activated amino-protected amino acids, which results in the addition of residues to the expanding peptide, one amino acid at a time. Each step of this process includes a deprotection reaction and a coupling reaction. Upon completion of the peptide, it is chemically cleaved from the resin. The peptide was purified by reverse-phase HPLC and its purity and molecular mass were confirmed using MALDI-TOF mass spectrometry. The primary structure was confirmed by LC/MS/MS.

### 4.5. Rat Tail Artery Smooth Muscle Bioassay

#### 4.5.1. Tissue Preparation

Wistar rats were asphyxiated using CO_2_ followed by cervical dislocation carried out in accordance with U.K. Animal Experimentation guidelines. The tail artery was prepared as described previously [10].

#### 4.5.2. Pharmacological Bioassay

Serial dilution was used to prepare solutions of the peptide and bradykinin (Sigma-Aldrich, St. Louis, MO, USA) using a 1 mM stock solution in Kreb’s buffer. The peptide dose responses were performed and recorded following perfusion of peptides at concentrations of 10^−9^–10^−3^ M into the 2 mL organ bath resulting in overall dose concentrations of 10^−11^–10^−5^ M for both baltikinin and BK. The changes in muscle tone were measured by the transducer, and the response of the tissue could be determined by comparison with spontaneous baseline contractions.

The collected data were analyzed using Graphpad Prism software (version 6.01, GraphPad Software, Inc., La Jolla, CA, USA, 2012) to construct the dose-response curve using a line of best fit algorithm. Responses were plotted as tension changes of contraction against the log of final molar concentration of peptide present in the organ bath. Data points were represented as mean values of contraction ± SEM, where *n* = 7. The software also allowed the EC_50_ values (concentration to elicit half the maximum response) of baltikinin and BK to be calculated.

## Abbreviations

MDPI	Multidisciplinary Digital Publishing Institute
DOAJ	Directory of open access journals
HPLC	High performance liquid chromatography
cDNA	complmentary deoxyribonucleic acid
BLAST	Basic Local Alignment Search Tool
NCBI	National Centre for Biotechnological Information
MALDI-TOF	Matrix-assisted laser desorption/ionisation, time-of-flight
MS	mass spectrometry
MS/MS	Tandem mass spectrometry
SEM	Standard error of the mean
TFA	Trifluoroacetic acid
PS	Peptide synthesiser
LC/MS/MS	Liquid chromatography coupled tandem mass spectrometry
